# Supplementary material for: Engineering macrophage responses through 3D scaffold microarchitecture
Source: Mater Today Bio. 2025 Sep 21;35:102328. doi: 10.1016/j.mtbio.2025.102328 (PMC12517081; doi:10.1016/j.mtbio.2025.102328)
Supplement: Multimedia component 1 [file mmc1.docx]

**Appendix A. Supplementary data**

**Engineering macrophage responses through 3D scaffold microarchitecture**

Chiara Martinelli^1*^, Srijan Chakraborty^1^, Giovanni Buccioli^1^, Matteo Vicini^1^, Claudio Conci^1^, Giulio Cerullo^2^, Roberto Osellame^2^, Giuseppe Chirico^3^, Emanuela Jacchetti^1#*^, Manuela Teresa Raimondi^1#^

^1^Department of Chemistry, Materials and Chemical Engineering “Giulio Natta”, Politecnico di Milano, Piazza L. da Vinci, 32, 20133 Milan, Italy

^2^Institute for Photonics and Nanotechnologies (IFN), CNR and Department of Physics, Politecnico di Milano, Piazza L. da Vinci, 32, 20133 Milan, Italy

^3^Department of Physics, Università di Milano-Bicocca, Piazza della Scienza, 3, 20126 Milan, Italy

**^*^**corresponding authors; chiara.martinelli@polimi.it, emanuela.jacchetti@polimi.it

^#^co-last authors

**
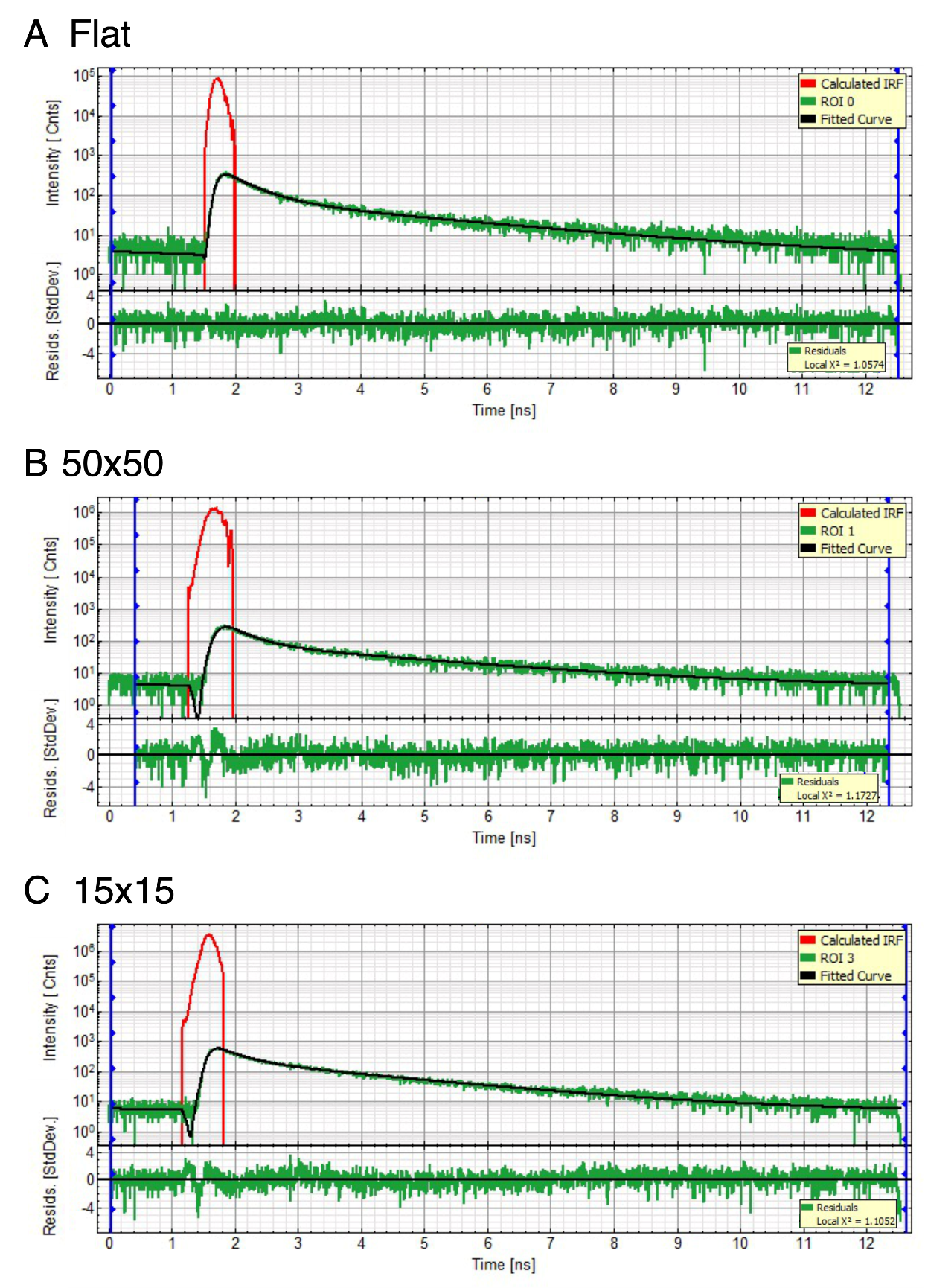
**

**Supplementary Figure 1. Representative fluorescence lifetime fitting curves.** (**A-C**) Representative fluorescence decay curves from FLIM measurements acquired on Flat, 50×50, and 15×15 scaffolds, respectively. Graphs were exported directly from SymPhoTime 64, the software used for data analysis. In each plot, the green line represents the fluorescence intensity decay measured in a single manually drawn cellular ROI. The black line corresponds to the fitted decay model, while the red line shows the instrument response function (IRF). Below each decay curve, the residuals of the fit (green) are shown, along with the reduced chi-squared (χ²) values indicating the goodness of fit.

**
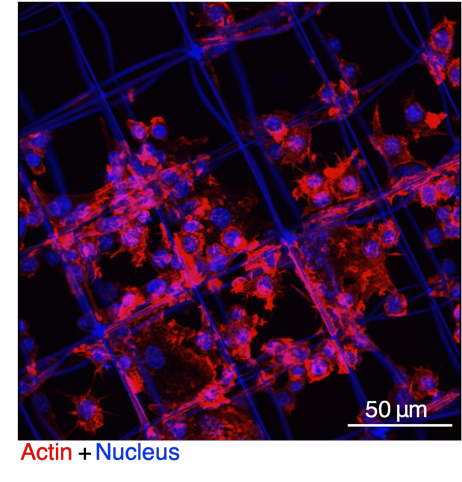
**

**Supplementary Video 1.** Representative 3D confocal laser scanning microscopy reconstructions of M0 macrophages cultured in the 50x50, stained for filamentous actin (red) and nuclei (blue). Due to the resin’s autofluorescence at the blue wavelengths, the scaffold is also visible in the blue channel alongside the cell nuclei.

**
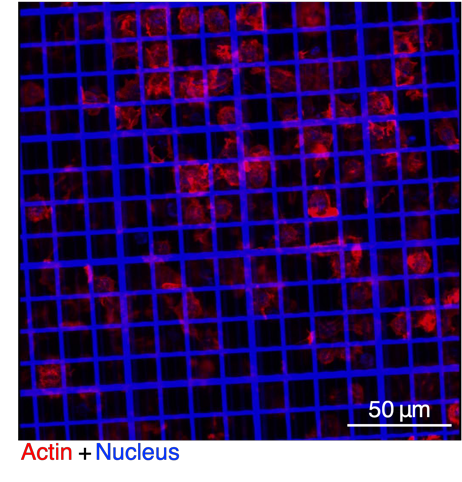
**

**Supplementary Video 2.** Representative 3D confocal laser scanning microscopy reconstructions of M0 macrophages cultured in the 15x15, stained for filamentous actin (red) and nuclei (blue). Due to the resin’s autofluorescence at the blue wavelengths, the scaffold is also visible in the blue channel alongside the cell nuclei.

**
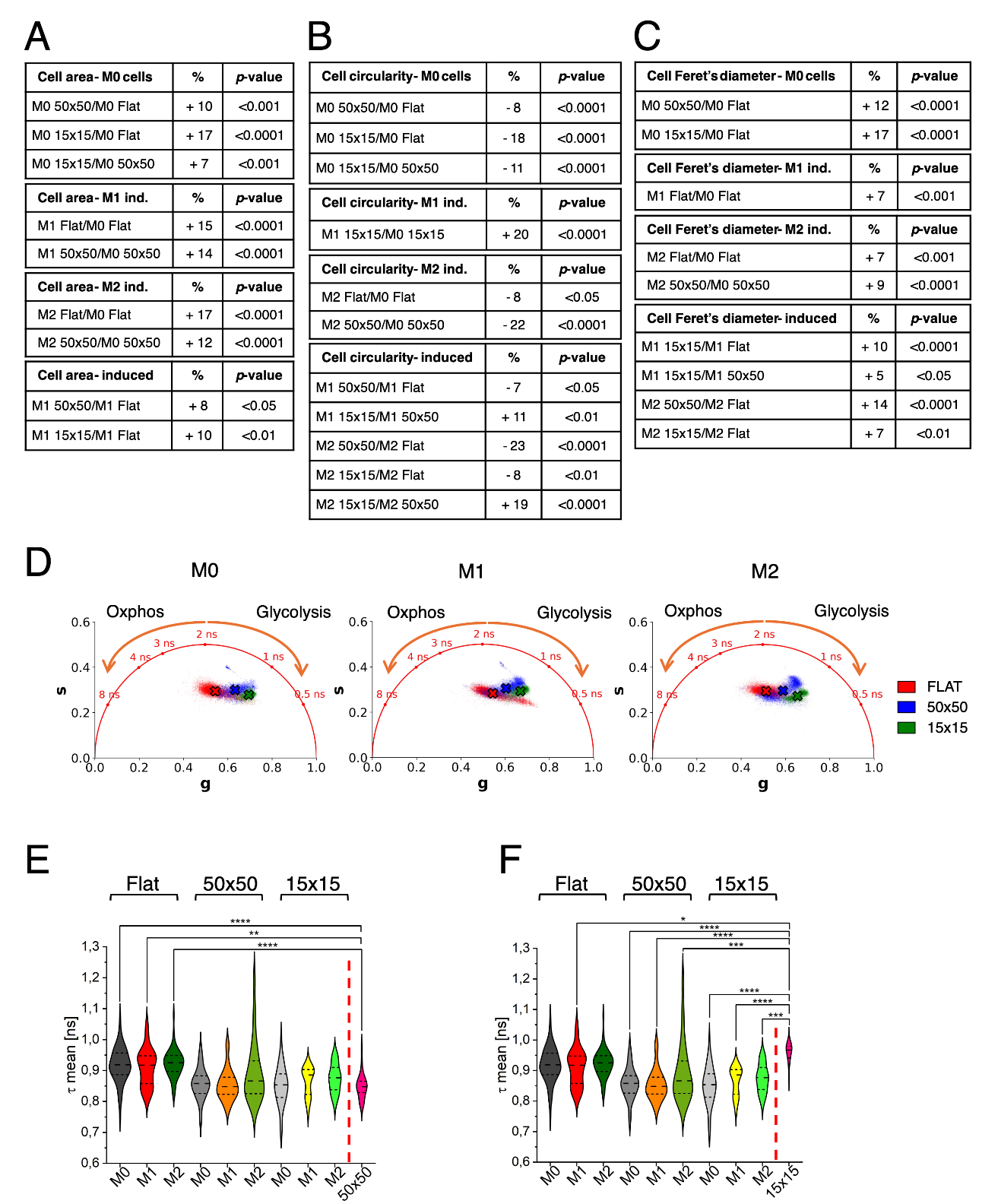
**

**Supplementary Figure 2.** (**A-C**) Quantification of morphological parameters: cell area (**A**), circularity (**B**), and maximum Feret’s diameter (**C**) for M0, M1, and M2 macrophages cultured on Flat, 50x50, and 15x15 substrates in terms of variations (%). **D**) Comparison of metabolic activity across the same conditions, as assessed by phasor FLIM analysis. The crosses in the phasor plots represent the centroids of the data clouds, corresponding to the average fluorescence lifetimes; a shift toward the right (shorter lifetimes) indicates a more glycolytic metabolic state. **E-F**) Summary of metabolic variations in M0, M1, and M2 macrophages across the three substrates, alongside the mean fluorescence lifetime values for the two scaffolds: 50x50 (**E**), and 15x15 (**F**). Samples 50x50 and 15x15 on the *X* axis, separated by the dotted red line, indicate the 50x50 and the 15x15 empty scaffolds, respectively. n ≥ 34 for each condition, n ≥ 16 for the scaffolds, *p-value < 0.05; **p-value < 0.01; ***p-value < 0.001; ****p-value < 0.0001. Collected n were obtained from at least three independent experiments.


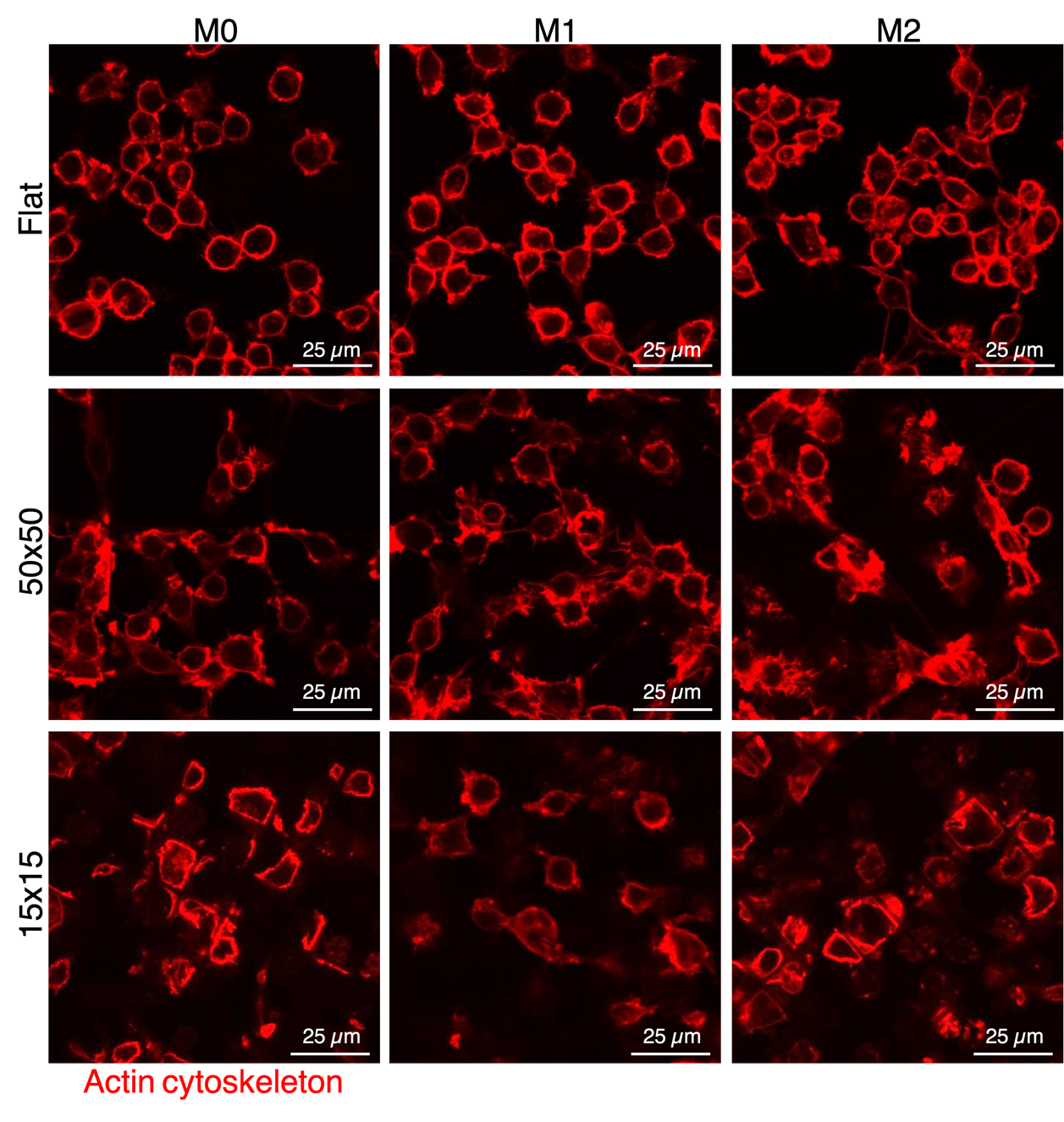


**Supplementary Figure 3. 3D microstructures modify morphology of macrophages in combination with chemical stimulation.** Representative confocal laser scanning microscopy images of M0, M1, and M2 macrophages cultured on Flat, 50x50, and 15x15 microstructures, and stained with Phalloidin-TRITC to visualize the actin cytoskeleton.

**
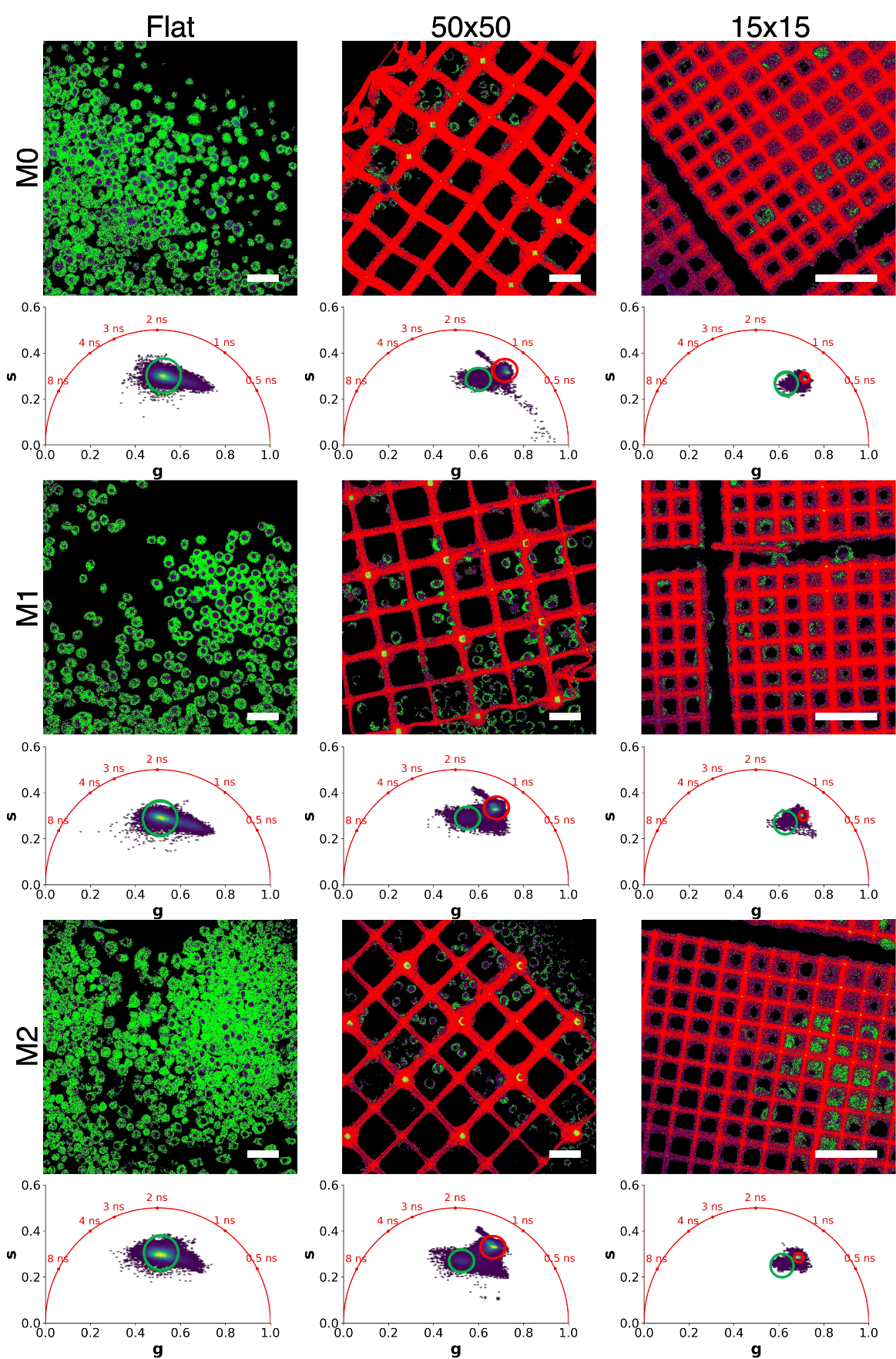
**

**Supplementary Figure 4.** Representative fluorescence lifetime imaging microscopy (FLIM) images (top) and corresponding phasor plots (bottom) of M0, M1, M2 macrophages cultured on the flat glass substrate and in the 50x50 and 15x15 microstructures. Classification of cells (in green) and scaffolds (in red) performed using corresponding circles on the phasor plot. Scalebar 40 µm.
